# Magnetoelectric Memory Based on Ferromagnetic/Ferroelectric Multiferroic Heterostructure

**DOI:** 10.3390/ma14164623

**Published:** 2021-08-17

**Authors:** Jiawei Wang, Aitian Chen, Peisen Li, Sen Zhang

**Affiliations:** 1 College of Science, Zhejiang University of Technology, Hangzhou 310023, China; wangjiawei@zjut.edu.cn; 2 Physical Science and Engineering Division, King Abdullah University of Science and Technology, Thuwal 23955-6900, Saudi Arabia; 3 College of Intelligence Science and Technology, National University of Defense Technology, Changsha 410073, China; 4 College of Liberal Arts and Sciences, National University of Defense Technology, Changsha 410073, China

**Keywords:** FM/FE multiferroic heterostructure, straintronics, volatile and nonvolatile, MTJ, magnetoelectric memory

## Abstract

Electric-field control of magnetism is significant for the next generation of large-capacity and low-power data storage technology. In this regard, the renaissance of a multiferroic compound provides an elegant platform owing to the coexistence and coupling of ferroelectric (FE) and magnetic orders. However, the scarcity of single-phase multiferroics at room temperature spurs zealous research in pursuit of composite systems combining a ferromagnet with FE or piezoelectric materials. So far, electric-field control of magnetism has been achieved in the exchange-mediated, charge-mediated, and strain-mediated ferromagnetic (FM)/FE multiferroic heterostructures. Concerning the giant, nonvolatile, and reversible electric-field control of magnetism at room temperature, we first review the theoretical and representative experiments on the electric-field control of magnetism via strain coupling in the FM/FE multiferroic heterostructures, especially the CoFeB/PMN–PT [where PMN–PT denotes the (PbMn_1/3_Nb_2/3_O_3_)_1−x_-(PbTiO_3_)_x_] heterostructure. Then, the application in the prototype spintronic devices, i.e., spin valves and magnetic tunnel junctions, is introduced. The nonvolatile and reversible electric-field control of tunneling magnetoresistance without assistant magnetic field in the magnetic tunnel junction (MTJ)/FE architecture shows great promise for the future of data storage technology. We close by providing the main challenges of this and the different perspectives for straintronics and spintronics.

## 1. Introduction

The growth of human civilization boosts the ravenous demand of data storage. Nowadays, the typical storage technologies include electrical recording, magnetic recording, and optical recording [1]. Among them, magnetic recording is dominated in the information storage, due to its comprehensive performance, most notably in non-destructive readout, large capacity, and high reading speed. Here, we focus the memory on the magnetic recording prototype.

Magnetic random access memory (MRAM) is one of the most promising candidates for the next-generation memory due to its high storage density and nonvolatile superiority, whose storage element form the fundamentals for spintronic devices. Prototype spintronic devices [2,3], i.e., spin valves and magnetic tunnel junctions, are sandwich architecture in which a nonmagnetic spacer is sandwiched by two ferromagnetic (FM) layers. The spacer is a conductive metal in spin valves, but an ultrathin insulator in magnetic tunnel junctions (MTJs) acting as a tunnel barrier. The relative magnetization alignment between the up and down FM layers determines the resistance of the device in both cases, corresponding to the tunnel magnetoresistance (TMR) effect [4] in MTJs and to the giant magnetoresistance (GMR) effect [5] in spin valves, respectively. The resistance is high when the magnetization alignment is antiparallel and is low when parallel. Therefore, changing the magnetization alignment, such as fixing one magnetic layer and rotating the other, could manipulate its resistance to accomplish “writing” operation in memory. 

**Figure 1:**
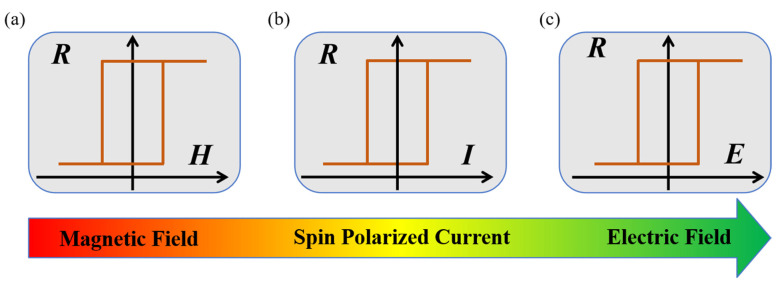
The sketch of magnetic recording prototype evolution. Writing data with (**a**) magnetic field, (**b**) spin polarized current, (**c**) electric field.

Historically, toggling the resistance states required classical magnetic field as intermediary. Writing data via magnetic field implies large power consumption in addition to complicated cell architecture (Figure 1a). The discoveries of the spin-transfer torque (STT) [6] and spin-orbital torque (SOT) [7] effect advanced the spintronics over the past two decades, in terms of density, energy dissipation, and scalability over the magnetic-assisted writing, in which a writing process is achieved by passing the spin current (Figure 1b). However, they still suffered from the shortcoming of energy efficiency, since the lowest switching energy via STT or SOT is limited to 10 fJ–100 fJ [8,9], which is two orders of magnitude larger than each switch in complementary metal-oxide-semiconductor (CMOS) transistor (<1 fJ). Regarding this, the quest for lower-power writing candidate using voltage or electric field rather than current to control magnetic properties [10] was spurred. 

The research was revived by the renaissance of the multiferroic compound in 2003 [11,12,13,14], in which several ferroic orders, for instance, the ferroelectric (FE) and magnetic orders, could coexist and couple together, thus generating the expectation of voltage or electric-field control of MRAM (Figure 1c). People initially paid attention to the single-phase multiferroics in which electric field could modify magnetic properties via magnetoelectric (ME) coupling. Unfortunately, most of these compounds show multiferroics at a cryogenic temperature. Recent researches display that genuine multiferroics with room temperature ferroic orders [15,16,17] is mostly present in complex compounds, which would be difficult to integrate with classical MRAM technology. In addition, the weak ME coupling in the single-phase multiferroics is far below the applicable requirement. In fact, most of single-phase multiferroics have a dominant antiferromagnetic (AFM) order and small remanent magnetization. It is still lacking in single-phase multiferroic simultaneously with a large magnetization and strong ME coupling at room temperature.

**Figure 2:**
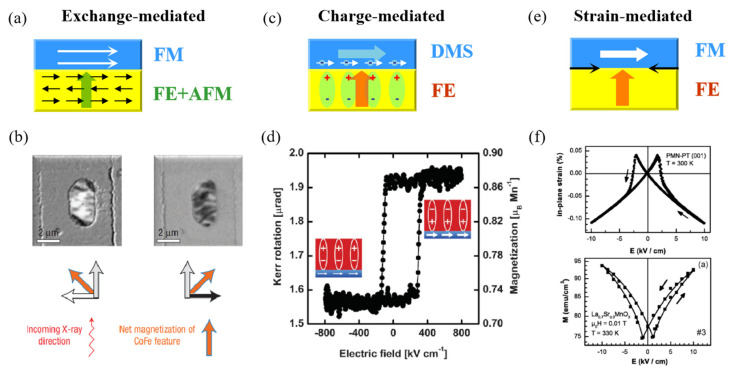
ME coupling through three main mechanisms in the FM/FE multiferroic heterostructure. (**a**) Exchange-mediated. (**b**) The representative work that the net in-plane magnetization of CoFe was controlled by the electric field at room temperature. (**c**) Charge-mediated. (**d**) The representative work that the nonvolatile electric field of Kerr rotation in LSMO/PZT heterostructure at 100 K. (**e**) Strain-mediated. (**f**) The representative work that the in-plane magnetization of La_0.7_Sr_0.3_MnO_3_ film was tuned by the strain of the PMN–PT FE substrate. Both of the in-plane strain and magnetization versus electric field curves show the butterfly-like shape. Reproduced with permission from reference [18,23,26].

The scarcity of single-phase multiferroics at room temperature spurs zealous research in the pursuit of composite systems combining a ferromagnet with FE or piezoelectric materials. So far, it has been recognized that artificial FM/FE multiferroic heterostructures are promising for applications due to the significant ME coupling at room temperature using the variety of room temperature FM and FE materials. Practically, three main physical mechanisms play the role in the ME coupling in such architecture, as demonstrated in Figure 2a–f. The first one involves the modifying exchange interaction between FE and FM or AFM orders, as illustrated in Figure 2a,b. The electric field first controls the direction of FE polarization, then has an effect on the magnetic order via exchange interaction, which is also confirmed in most single-phase multiferroics [18,19,20,21,22]. The second one is the influence of FE polarization direction on the ferromagnet’s interfacial electronic structure (Figure 2c,d). This influence occurs through two kinds of electronic effects: (a) field effect (i.e., depletion and accumulation of charge in the FM/FE heterostructure interface to screen the polarization charges of the FE); and (b) modification of the orbital hybridization around the interface between the ferromagnet and the FE [23,24]. Such electronic effects can generate two different magnetic states at polarization remanence, depending on the direction of the polarization. Both above mechanisms are closely related to the interface between FE and FM. Finally, an indirect mechanism via strain coupling could be effective in the FM/FE heterostructures (Figure 2e,f) [25,26,27,28,29,30,31,32,33,34]. Since every FE is also piezoelectric, the applied voltage across the heterostructure yields a modification in the dimensions of the FE through the converse piezoelectric effect. The strain will then be transferred to adjacent ferromagnet to tune its magnetic properties via the converse magnetostriction effect. In principle, strain coupling acts over the whole FM film, because strain is a long-range order parameter that can affect the thickness of magnetic film up to several hundred nanometers [35].

The literatures describing composite ceramics combining piezoelectrics and magnetostrictive components are not covered here. Rather, we review the progress on (a) artificial FM/FE multiferroic heterostructure in which the ME coupling occurs via strain, and (b) the ME memory constructed on this structure. It should be emphasized here that three types of ME memories are rather similar but not included here. The first one is the ME memory achieved by voltage-controlled magnetic anisotropy (VCMA) effect [36,37]. These MTJs have similar architecture as those used in STT devices, offering the advantage of a fast technological adoption. The switching mechanism is centered around these electronic effects. However, such VCMA-assisted MTJs still need a magnetic field to determine the switching direction. It is difficult to achieve the voltage control of switching direction without a combination of other effects [8]. The second one is the spin valves constructed on the single-phase multiferroic material BiFeO_3_ [20,22,38,39,40]. The ME mechanism in such a structure occur firstly through the exchange interaction between FE and AFM orders of the BiFeO_3_ layer, and then transfer to the spin valve via the AFM/FM exchange interaction, rather than the strain effect. These kind of devices are easily destroyed due to the unstable interface and irreversible oxidation of the ferromagnetic layer under the switching electric field. The last type is the FE MTJ [41,42,43,44], in which a FE-insulating spacer is sandwiched by two FM electrodes. The electric field influences the FE polarization direction on the spin filter effect of the FM layers alignment, thus achieving a four-state tunneling resistance. Unfortunately, all of the FE MTJs can only work at low temperatures. There is still a long way to go for application at room temperature. More information on FE MTJ can be found in the reviews [45,46].

Multiferroic ME switching driven by FE switching is considered as the most energy-efficiency avenue in nanoscale manipulation at room temperature. The modern computing devices based on FM/FE multiferroic heterostructure offer the advantages of high compaction, low power density, and short time delay [47]. Compared with other ME switching mechanisms, strain coupling driven by FE switching is superior in its switching speed, switching reliability, and capability of 180° switching, especially in its ultralow heat dissipation down to attoJoule, despite its shortcoming of size scalability [48]. ME memory based on strain-mediated multiferroic heterostructure is promising in energy efficiency and heat dissipation, and the great superiority can be displayed when such memory is closely integrated with logic. This paper historically reviews the development of ME memory achieved in a strain-mediated FM/FE multiferroic heterostructure. As the basis for devices discussed in later sections, we first introduce the theories and experiments of electric field influence on macroscopic magnetic anisotropy and moments via strain coupling in a single FM film, especially in the amorphous CoFeB film, which is frequently used in the spintronic devices. The key features in the electric-field control of magnetism, such as “nonvolatile” and “reversible”, is realized step by step via FE domain engineering. We then present recent strain-driven electric-field control of GMR and TMR in the different sections on the basis of such architectures. GMR is first discussed due to the fabrication superiority. Challenges and perspectives for this field are also provided at the end of the review. 

## 2. Electric-Field Control of Magnetic Anisotropy

In an FM system, macroscopic magnetic properties involve anisotropy, moment, order, domain, Curie temperature (*T*_C_), spin polarization, and exchange interaction with other layers. In fact, all aspects can be influenced by strain, and typical outstanding works have been accomplished in different architectures. We focus the attention on the magnetic anisotropy manipulation via strain from the FE materials in the FM/FE multiferroic heterostructures in this chapter.

Anisotropy means the magnetism is not identical in all directions. Some special axis of a sample lies along some fixed directions. Usually, the magnetic easy axis (i.e., the easy axis of magnetization) lies in the direction in which the total free energy *F* is minimized. When there is no external magnetic field, *F* includes the demagnetization energy *F*_demag_, magnetocrystalline anisotropy energy *F_mc_* if the sample is crystallized, and the surface (or interface) magnetic energy *F*_surf_. The magnetoelastic energy *F*_me_ should be included if the ferromagnet is in contact with a FE, since voltage-induced strain effects impact the magnetic properties. Moreover, *F*_surf_ will depend on voltage when electronic effects appear at the interface [49]. 

**Figure 3:**
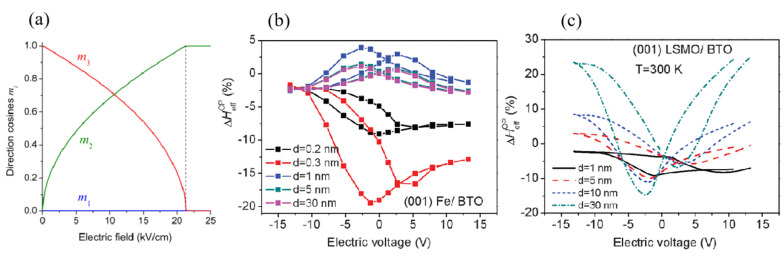
Calculation of voltage-induced changes in magnetic anisotropy. (**a**) Electric field rotates the direction of magnetic easy axis 90° in the Ni layer. Calculation combining both strain and electronic effects via the electric field dependence of out-of-plane anisotropy field for Fe/BTO heterostructure (**b**), and LSMO/BTO heterostructure (**c**). Reproduced with permission from reference [49,51].

Theoretically, for a system combining an FE with a large electrostriction coefficient and a soft ferromagnet with a strong piezomagnetic coefficient, a large *F*_me_ can be produced under a modest voltages to change the magnetic easy axis’ direction. As illustrated in Figure 3a for the Ni/(PbZn_1/3_Nb_2/3_O_3_)_1-x_-(PbTiO_3_)_x_ (Ni/PZN–PT) system [50], Pertsev [51] calculated that applying an electric field of 20 kV/cm across the heterostructure could change the direction of magnetic easy axis and generate 90° rotation of the magnetization in the Ni layer. CoFe_2_O_4_/PZN–PT [51] and many other combinations [52] showed the similar results. For various systems, the conjunct influences of voltage-induced field effect and strain effect as a function of FM layers’ thickness [see the applied voltage dependence of the out-of-plane anisotropy field in Figure 3b for Fe/BaTiO_3_ (Fe/BTO) and Figure 3c for La_0.__7_Sr_0.__3_MnO_3_/BaTiO_3_ (LSMO/BTO)] were carefully analyzed by Hu et al. [51]. Both of the Fe/BTO and LSMO/BTO heterostructures show a relatively symmetric response to the voltage with a thick FM layer, suggesting the dominant role of the strain effect. Comparatively, the responses become more asymmetric due to the increasing influence of the interfacial field effect below a critical thickness (a few nanometers for the LSMO layer and a few angstroms for the Fe layer). These calculations thus reflect that nonvolatile voltage control of magnetization requires extremely high-quality thin FM layers to be grown onto FEs.

**Figure 4:**
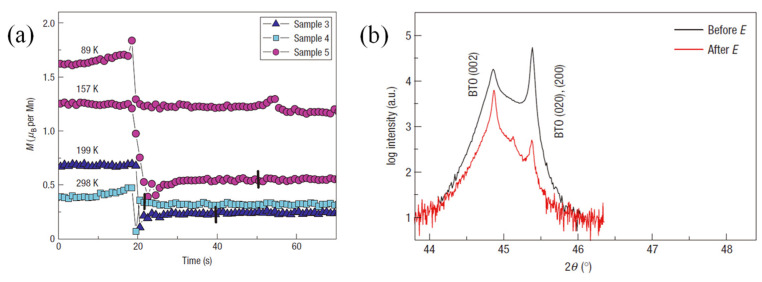
Representative experimental work of strain-driven electric-field control of magnetization in the La_0.67_Sr_0.33_MnO_3_/BaTiO_3_ heterostructure. The nonvolatile electric-field control of magnetization (**a**) and corresponding FE domain switching (**b**) in the La_0.67_Sr_0.33_MnO_3_/BaTiO_3_ heterostructure. Reproduced with permission from reference [25].

Eerenstein et al. [25] first reported the experimental work in 2007, in which the La_0.67_Sr_0.33_MnO_3_ film with 40-nm thickness was epitaxially grown onto the BaTiO_3_ substrate, and nonvolatile electric-field control of magnetization was obtained, as shown in Figure 4a. The mechanism is understood as follows. The tetragonal-phase BaTiO_3_ single crystal exhibits two types of FE domains orientations (c-domain and a-domain) at room temperature. The applied electric field across the sample would switch the FE domain and develop into a single-phase BaTiO_3_, accompanying with generation of in-plane local stress, which is transferred to the La_0.67_Sr_0.33_MnO_3_ film, as shown in Figure 4b. Since the domains switching in BaTiO_3_ are irreversible (i.e., nonvolatile), the electric-field control of magnetization is also irreversible (i.e., nonvolatile). Although the electric-filed control of magnetization is irreversible (reset is complicated) and repeatedly poling with large electric field can easily fatigue and break the BaTiO_3_ crystal, Eerenstein et al. first introduced the idea of nonvolatile electric-field control of magnetization by strain from the switching of local FE domains, which has application potential. 

Thiele et al. first demonstrated the obvious evidence for the electric-field control of magnetic anisotropy via a strain in the multiferroic heterostructure combining the (001)-oriented PMN–PT [where PMN–PT denotes (PbMn_1/3_Nb_2/3_O_3_)_1-x_-(PbTiO_3_)_x_] single crystal with the epitaxial La_0.7_A_0.3_MnO_3_ (A = Sr, Ca) film [26]. They measured the room temperature in-plane strain versus the electric field (S-E) curve of the PMN–PT substrate, which displayed butterfly-like behavior in Figure 2f. Interestingly, the in-plane magnetization to the electric field stimulation in Figure 2f also had a butterfly-like shape. The similarity in Figure 2f suggests the close connection between the in-plane strain from the PMN–PT crystal and the magnetization from the LSMO film. This ME coupling is considered given how the PMN–PT single crystal transfers the in-plane biaxial strain onto the epitaxial LSMO film and varies the angle and length of the Mn-O bond, thus modifying the *T_c_* and magnetization of the LSMO film. Notably, different from the work in the La_0.67_Sr_0.33_MnO_3_/BaTiO_3_, the reversible strain of the PMN–PT single crystal generates the reversible magnetization manipulation in the LSMO film without a complicated reset process, offering the advantages in terms of easy operability in practical application, thus initiating plenty of analogous work in the artificial FM/FE multiferroic heterostructures, such as Fe/BaTiO_3_ [53,54], FeCoV/PZN–PT [55], CoFe_2_O_4_/PMN–PT [56], NiFe_2_O_4_/PMN–PT [57], FeGaB/PZN–PT [58], Fe_3_O_4_/PZT and Fe_3_O_4_/PMN–PT [59], FeBSiC/PZT [60], ZFO/PMN–PT [61], Ni/BaTiO_3_ [62], CoFe/BaTiO_3_ [63], Fe–Ge/BSPT, Fe–Ge/PZT, and Ni/PZT [64], Ni/PMN–PT/Ni [65] as well as many other combinations. 

As most FE materials display a butterfly-like shape and S-E curves under the bipolar electric field, as shown in Figure 2f, the magnetic responses of these strain-mediated heterostructures will show the butterfly-like shapes as shown in Figure 2f. The “volatile” characteristic is the biggest problem in these ME behaviors, i.e., the modification of magnetic responses manipulated by electric field vanishes when the applied electric field returns to zero, since the strain restores to the initial value under zero electric field, which is the drawback for energy conservation. In fact, “nonvolatile” and “reversible” are two key factors associated with the ME memory. How to achieve the electric-field control of magnetization at room temperature via strain coupling combining the nonvolatile response as the results in La_0.67_Sr_0.33_MnO_3_/BaTiO_3_ [25] and absolute reversibility as the results in LSMO/PMN–PT [26] is an important and interesting issue. 

**Figure 5:**
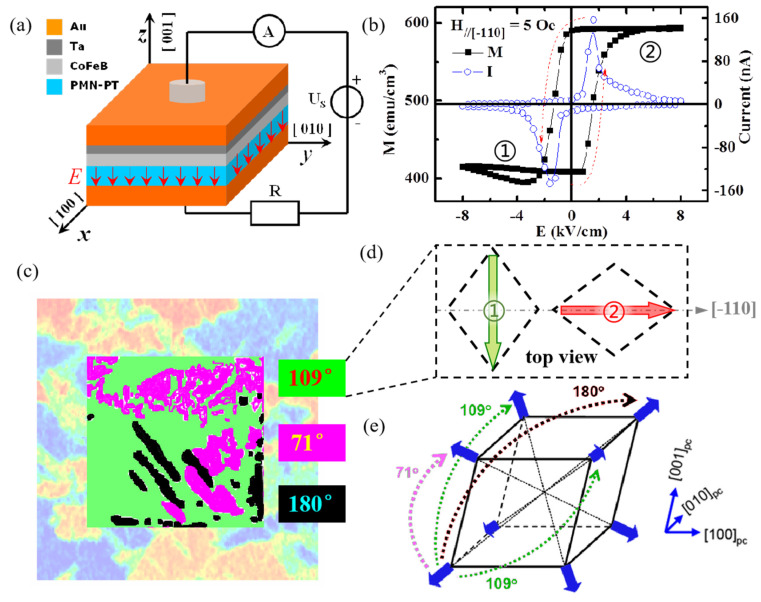
The nonvolatile and reversible electric-field control of magnetization in the CFB/PMN–PT (001) heterostructure. (**a**) Scheme of the sample and experiment configuration. (**b**) Electric-field control of the magnetization (solid square) and polarization current (open circle) recorded simultaneously, in which magnetization of state 1 and 2, respectively, corresponds to the lattice deformation of state 1 and 2 in (**d**). (**c**) Three types of FE domain switching, namely 71° (purple), 109° (green), and 180° (black) in the (001)-cut PMN–PT single crystal, measured by piezoresponse force microscopy. (**d**) Top view of the rhombohedral lattice deformation in the 109° domain switching. (**e**) The corresponding changes of the FE polarization direction for the three types of FE domain switching in each rhombohedral lattice. Reproduced with permission from reference [69].

**Figure 6:**
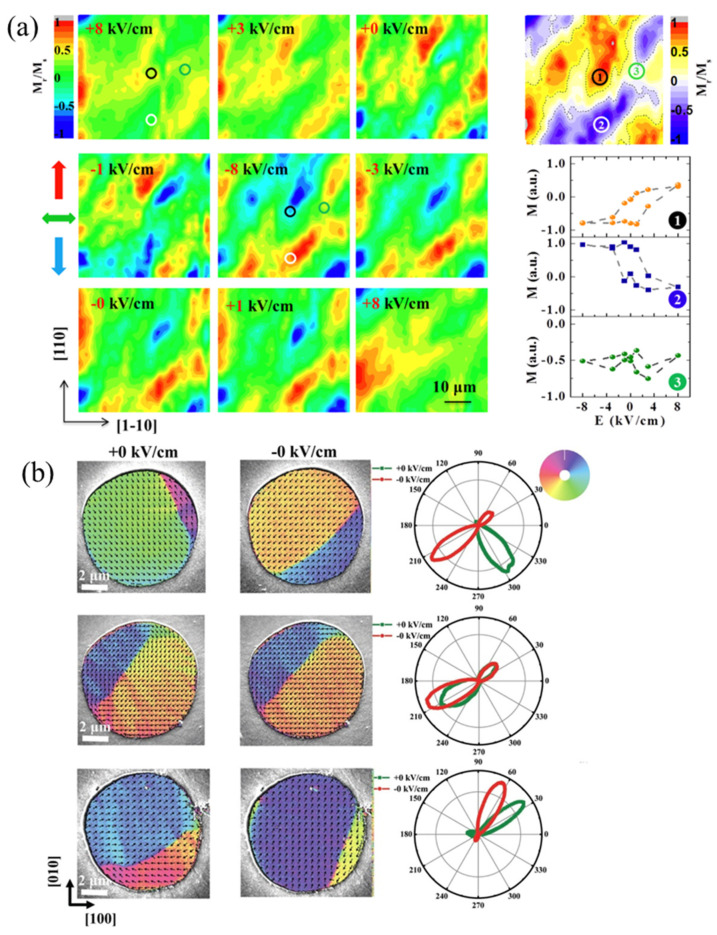
Intrinsic spatial inhomogeneity of ME behavior in the CFB full film and discs on the (001)-cut PMN–PT substrate. (**a**) Scanning Kerr microscopy maps of *M*_r_*/M*_s_ in a 50 × 50 μm^2^ area under different electric fields. The difference of signal and the local electric-field control of magnetization (M-E) curves in the different regions are collected on the right. (**b**) Scanning electron microscopy with polarization analysis (SEMPA) images of different discs are marked as type I (top), type II (middle), and type III (bottom), respectively. The right side are the polar plots analysis indicates the distribution of magnetization directions in the left SEMPA images, in which the red/olive curves represent the remanent states of the negatively/positively polarized cases. Reproduced with permission from reference [73,74].

FE materials have domains to reduce the depolarization field, and domain switching occurs under certain external electric field [66]. For the case of the FE materials with rhombohedral phase such as PZN–PT, PMN–PT, and BiFeO_3_, there are three types of FE domain switching, namely 71°, 109°, and 180° domain switching [67,68]. In fact, nonvolatile strain coupling is closely related with the FE domain switching type, which is different from the nonvolatile electronic effects. The first representative work was reported by Zhang.et al. [69], in which amorphous Co_40_Fe_40_B_20_ (CFB) film with a 20 nm thickness was deposited onto the PMN–PT (001) substrate, as shown in Figure 5a. The nonvolatile and reversible control of magnetization under bipolar electric field is observed in Figure 5b. A detailed study on the PMN–PT substrate by in-situ piezoresponse force microscopy (PFM) and reciprocal space mapping (RSM) techniques evidenced the existence of three types of FE domain switching (Figure 5c,e). Considering the positive magnetostriction coefficient of the CFB film and the in-plane deformation of rhombohedral PMN–PT lattice, as shown in Figure 5d, the nonvolatile (volatile) ME behavior originated from the 109° (71° or 180°) switching induces the nonvolatile (volatile) strain [70]. The importance of the amorphous FM film should be emphasized here, since the nonvolatile or volatile strain could totally transfer onto the isotropic CFB film via a magnetoelastic interaction. Comparatively, the ME behavior on the crystalline FM film, such as the polycrystalline film, reported by Zhang et al. [71], or the epitaxial oxide film [26,56,72], will experience the competition between magnetocrystalline anisotropy energy *F*_mc_ and magnetoelastic energy *F*_me_, resulting in complicated medium ME behavior. Interestingly, by using in-situ scanning Kerr microscopy (SKM) and scanning electron microscopy with polarization analysis (SEMPA), Li et al. [73] and Ba et al. [74] further evidenced the intrinsic spatial inhomogeneity of FE switching on the mesoscale PMN–PT substrate, thus leading to the coexistence of loop-like (nonvolatile) and butterfly-like (volatile) ME behavior in the full CFB films and CFB mesoscopic discs, respectively, grown onto the PMN–PT (001) substrate, as shown in Figure 6a,b. It should be mentioned that the work of Liu et al. [75] demonstrated the (001)-cut PMN–PT phase-dependent ME behaviors in this composite system. The abundant structure phase in the PMN–PT single crystal provides a path for choosing the appropriate tuning behavior, i.e., loop-like, butterfly-like, or mix ME behavior, which paves the way for designing ME devices through domain engineering. 

**Figure 7:**
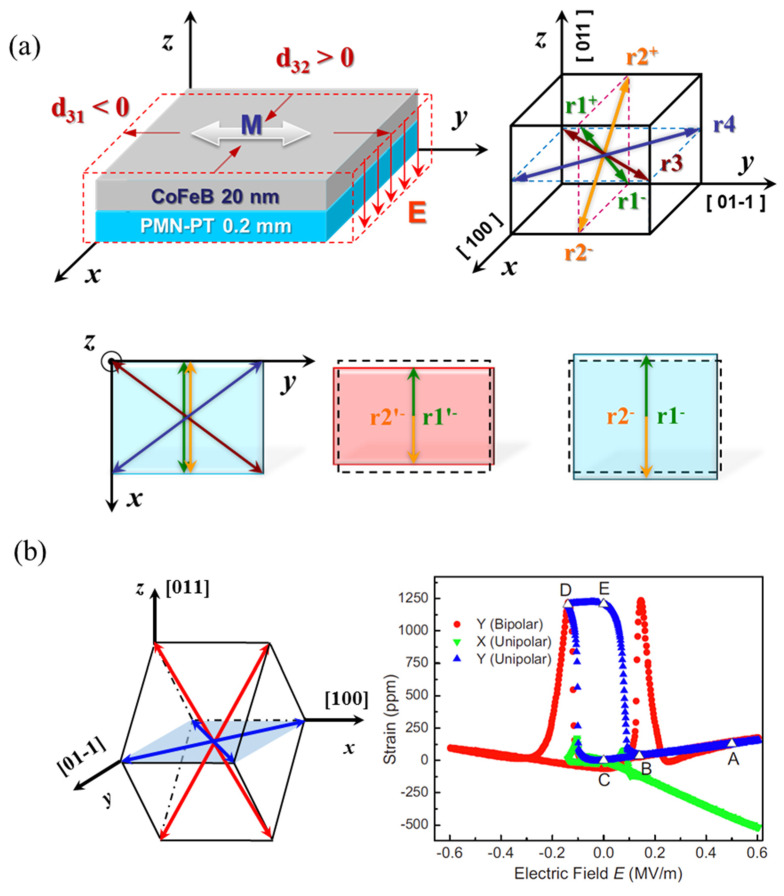
The anisotropic in-plane piezostrain and domain switching in the (011)-cut PMN–PT single crystal. (**a**) Sketch of the crystal structure, polarization of the (011)-cut PMN-PT, and the corresponding in-plane deformation under electric fields. (**b**) The polarization directions configuration for (011)-cut PMN–PT and in-plane piezostrains along the *y* and *x* direction. Reproduced with permission from reference [76,79].

The investigation on the CFB/PMN–PT (001) heterostructure reveals the nonvolatile and reversible electric-field control of magnetization by combining of the absence of magnetocrystalline anisotropy and the existence of the 109° FE domain switching. However, the complex of the structure phase in the (001)-cut single crystal leads to uncertainty of the FE domain switching type, generating unpredictable ME behavior, which is a drawback in the practical application of this structure. Comparatively, the FE domain switching is simpler in the (011)-cut PMN–PT single crystal, as shown in Figure 7a [76], although the FE domain switching is still inhomogeneous on the mesoscale substrate [77,78]. Moreover, the anisotropic in-plane piezoelectric coefficients, *d*_31_ and *d*_32_, is conductive to the generation of larger in-plane strain, serving an elegant platform to fulfill large and predictable ME behavior at room temperature, as well as promising ME devices. Zhang et al. [76] showed that the intensive in-plane anisotropic piezostrain could rotate the in-plane easy axis of the amorphous CFB film 90° reversibly, and that the relative change in Δ*M*/*M* is much larger than those in the (001)-cut PMN–PT. However, the ME behavior shows the “volatile” feature. By aiming to combine the advantages of “nonvolatile” and “reversible” in the architecture based on the (011)-cut PMN–PT or PZN–PT, Wu et al. [79], Wang et al. [80], and Liu et al. [81] reported an asymmetrical electric field applied avenue illustrated in Figure 7b, which is different from the bipolar electric field applied in Zhang et al.’s work. Here, the bipolar electric filed is the one applied between symmetric positive and negative filed, and the maximum value is larger than coercive field. Their efforts offer a new strategy for achieving a giant, nonvolatile, and reversible electric-field control of magnetization via strain coupling in the FM/FE heterostructures, which would be employed in the ME memory design. So far, FM/FE heterostructures have become a unique playground, not only for studying the strain-driven electric field manipulation novel physical effects, such as electric-field control of exchange bias [82,83], RKKY interaction [84], and skyrmions [85,86], but also for exploiting the related ME devices. 

## 3. Electric-Field Control of Giant Magnetoresistance

GMR (defined as (*R*_ap_*−R*_p_)/*R*_p_, in which *R*_ap_ denotes the resistance in the antiparallel state, *R*_p_ denotes the resistance in the parallel state) occurs in sandwich or multilayer architectures with alternating nonmagnetic metallic and FM layers [4]. The resistance depends on the alignment of the magnetization of the two FM layers. Both of current-perpendicular-to-plane (CPP) and current-in-plane (CIP) geometries are available for the GMR effect, whose sign and amplitude are determined by the spin-dependent scattering times at their interfaces and in the different layers [87]. Experiments have evidenced that the GMR from CPP geometries is much larger than the CIP geometries, but the main difficulty is from the microfabrication techniques [88].

GMR architecture becomes more elaborate in spin valves, in which one of the FM electrodes has exchange interaction with its adjacent antiferromagnet [89]. The magnetic hysteresis loop of this FM electrode enlarges and shifts in one magnetic field direction, owing to the exchange bias effect. The corresponding device can then be operated around the zero magnetic field in a narrow range, because the magnetization of the pinned layer (i.e., exchange-biased electrode) cannot be easily rotated, but the magnetization of the free layer (i.e., the other electrode) can be tuned by a modest magnetic field.

**Figure 8:**
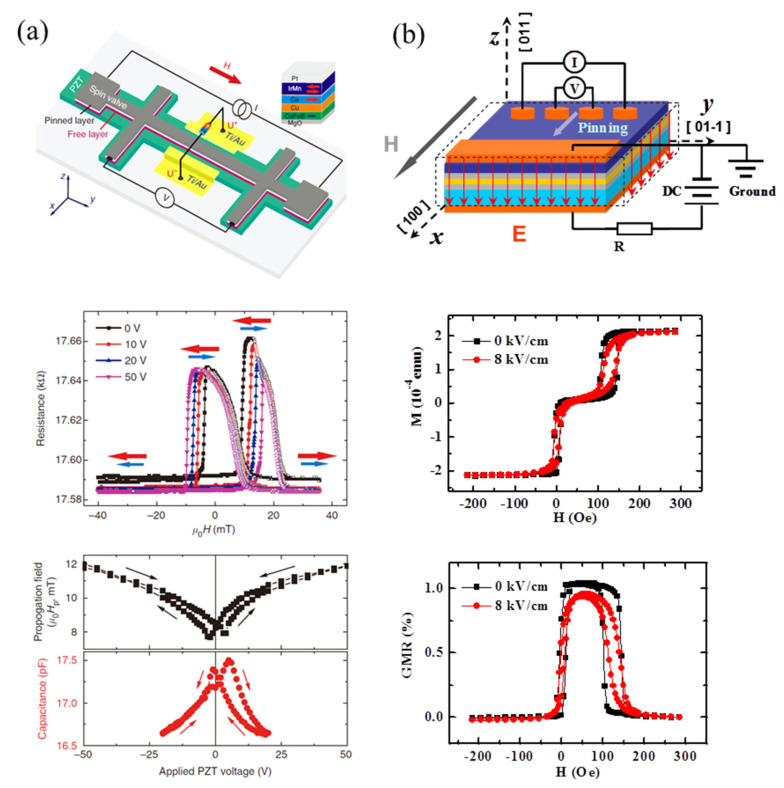
Strain-driven electric-field control of GMR. (**a**) Configuration of electric-field control of GMR in Co/Cu/CFB spin valve adjacent to the PZT layer. The reversible effect was shown as the voltage dependence of magnetic propagation field and capacitance in the bottom panel. The blue and red arrow in the middle panel denote the magnetization orientations of the CFB and Co layers, respectively. (**b**) Configuration of electric-field control of GMR in CFB/Cu/CFB spin valve on the (011)-cut PMN–PT substrate. The corresponding electric-field control of magnetization and GMR with applied magnetic field along the [100] direction. Reproduced with permission from reference [90,91].

Lei et al. [90] first reported the representative work on the electric-field control of GMR via strain coupling, in which a PZT FE film was used to realize the voltage control of magnetic anisotropy of the free CoFeB layer (Figure 8a) and the spin valve. Exchange bias in the GMR curve is present due to the existence of an AFM IrMn layer in contact with the adjacent FM Co layer. Both of the magnetic propagation field (*H*_p_) and the capacitance indicate butterfly-like dependence with voltage applied to the PZT, clearly confirming the nature of the strain coupling mechanism.

The spin valve on the PMN–PT (011) shows similar features to the curve on the PZT (Figure 8b from reference [91]), and the electric-filed control of GMR can be evidenced from the anisotropy change in the CFB free layer. The achievement of these devices proves challenges and requires the individual optimization of (a) the GMR’s amplitude and the modification of GMR, and (b) designing nonvolatile and reversible electric-field control of GMR.

## 4. Electric-Field Control of Tunneling Magnetoresistance

MRAM is more possible to be achieved in the MTJ structure, owing to the higher magnetoresistance ratio (Δ*R*/*R*) in the TMR than the GMR. In the MTJ architecture, two FM electrodes sandwich a very thin insulator as a tunnel barrier. All of the MTJs are CPP geometry, whereby the current is tunneled across the barrier from one FM electrode to the other. The same as the GMR effect, the TMR is also decided by the alignment of the magnetization of the two FM electrodes. The value of TMR relies on (a) the decay rates of the wave functions in the tunnel barrier, and (b) the spin polarization of the density of states at the interfaces between both electrodes and the barrier [92]. 

The proposal of electric field controlling of MTJs was set forth in pursuit of low-power consumption [36,37,92,93,94], instead of conventional magnetic field or STT with excessive high electric current density [95]. The discovery of multiferroic materials revived the ME research, including the electric-field control of TMR. As mentioned of FE MTJ in the introduction section, the employment of ultrathin FE or multiferroic layers as a tunnel barrier is one scheme to achieve ME control of TMR. However, there is still a long way to go for the application, due to the assurance that the barrier materials maintain FE at the extremely low thickness as a tunnel barrier [96] and the cryogenic operation temperature. Alternatively, an avenue of MTJ stacks grown onto the FE single crystal has been proposed theoretically [94,97,98]. Through strain-mediated ME coupling, electric field, rather than currents controlled by TMR, can be realized by manipulating the magnetization direction of the FM free layer. Therefore, this scheme proposed an ultralow-power dissipation, ultrahigh storage capacity, and high-speed MRAM device [94]. Meanwhile, the read and write cells are separated in the three terminal design of this device [99], and the voltage is not directly applied onto the MTJ to protect it. 

**Figure 9:**
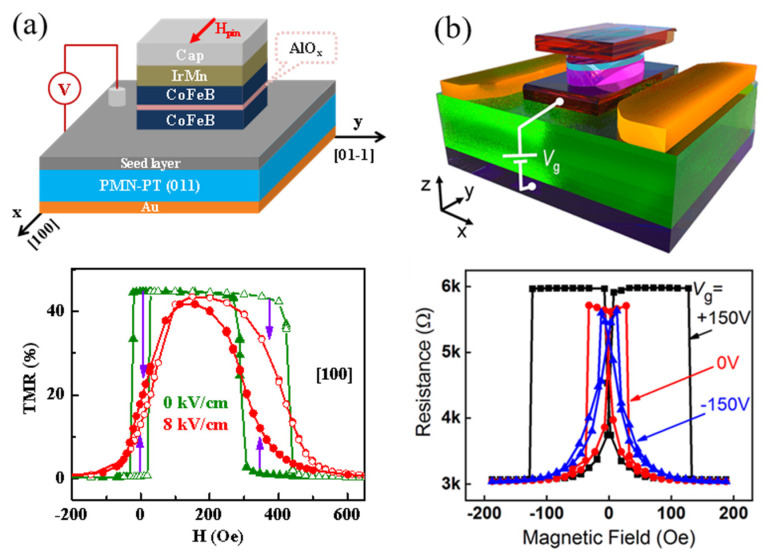
Strain-driven electric-field control of TMR. Volatile electric field manipulation of the resistance in (**a**) AlO_x_-based MTJ on the (011)-cut PMN–PT and (**b**) MgO-based MTJ on the (001)-cut PMN–PT. The employment of the AFM pinning layer in (**a**) shifts the resistance loop in one magnetic field direction. Reproduced with permission from reference [100,101].

The progress of experimental work on the MTJ/FE structure is slow due to the complicated fabrication process of MTJ which requires high-quality multilayer films and several steps of microfabrication. Until the year 2014, Li et al. [100] successfully fabricated CoFeB/AlO_x_/CoFeB MTJ with a TMR ratio of 45% on the PMN–PT (011) FE substrate. As shown in Figure 9a, the TMR curves had a remarkable change with electric fields. Importantly, the TMR was modulated by electric field at zero magnetic field with a modulation of 15%, which is attributed to the 90° rotation of the free layer’s magnetization in MTJ. This reversible electrical modulation of resistance in MTJs gives a promising approach to realize ME memory. Furthermore, Zhao et al. [101] deposited MgO-based MTJ on the PMN–PT (001) FE substrate, as shown in Figure 9b. An annealing process was performed to enhance TMR, and a TMR ratio about 90% was obtained. Electric fields also had a striking effect on the TMR curves, suggesting an electrical modulation of TMR in the MTJ/PMN–PT structure. This work shows that the annealing process does not affect the strain-mediated ME coupling. A significant difference in those two works is the use of the AFM pinning layer, which is exchange coupled to one of the FM electrodes. The exchange bias effect shifts the magnetic hysteresis loop in one magnetic field direction, producing the manipulation without the assistant magnetic field in this device, as shown in Figure 9a.

**Figure 10:**
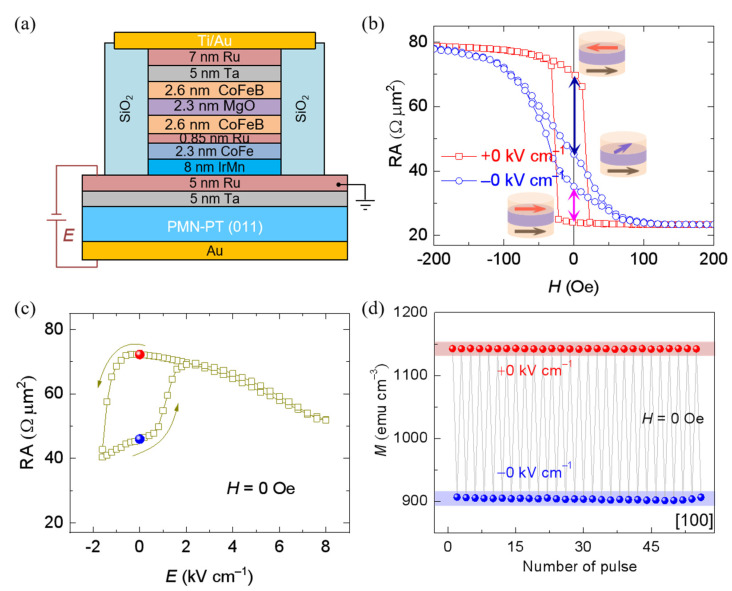
Strain-driven nonvolatile electric-field control of TMR. (**a**) Schematic of the MTJ stack on (011)-cut PMN–PT FE substrate. (**b**) The corresponding TMR curves at ±0 kV cm^−1^ after applying 8 kV cm^−1^ and −1.6 kV cm^−1^, respectively. Nonvolatile electric-field control of the resistance (**c**) and magnetization (**d**) in MTJ by applying asymmetric electric fields. Reproduced with permission from reference [102].

However, a volatile ME control of TMR is still used in both above works, which cannot solve the problem of huge energy consumption. Regarding this, using the nonvolatile piezostrain in the (011)-cut PMN–PT FE substrate by applying asymmetric electric fields [79,80,81], Chen et al. [102] fabricated MgO-based MTJs onto the PMN–PT (011) FE substrate with a TMR ratio up to 235% (Figure 10a). Owing to 90° rotation of the magnetic easy axis of the free layer driven by piezostrain of the PMN–PT substrate, the TMR curves at +0 kV cm^−1^ after applying 8 kV cm^−1^, and −0 kV cm^−1^ after applying −1.6 kV cm^−1^, are distinctly different in Figure 10b, suggesting a nonvolatile manipulation. Moreover, a nonvolatile electric-field control of TMR and magnetization in MTJ without an assistant magnetic field can be seen clearly in Figure 10c,d. The loop-like behavior in Figure 10c demonstrates that two resistance states at a zero electric field after exerting asymmetric electric fields can encode information, which represents a crucial step towards ME memory with magnetic reading and electric writing. 

As mentioned before, the resistance of an MTJ is closely related to the magnetization alignment of the two FM electrodes. A large relative angle between the up and down FM magnetization gives a high resistance of MTJs. The largest resistance change happens between the parallel and antiparallel magnetization configuration of the MTJ. Thus, the magnetization of the free layer needs to be reversibly switched between 0° and 180°. Furthermore, 180° magnetization switching is important for distinguishing the TMR value if MTJ is used to read out the information. The previous work has demonstrated the 90° magnetization rotation driven by the in-plane anisotropic piezostrain of PMN–PT. However, one-step 180° magnetization switching of a magnetic layer is forbidden due to the conservation of time-reversal symmetry [35]. The proposals of achieving 180° magnetization switching can be classified as three main types [48]. First, dipolarly coupled-nanomagnet-assisted 180° magnetization switching achieved in Co nanomagnet grown on PMN–PT (001) [103]. The second type is shape engineering [104,105,106], including nanomagnets with a special shape, such as a flower shapes [104], with four-fold magnetic anisotropy or nanoislands separated by modest distance [107]. The final avenue can be theoretically achieved by applying a nanosecond-long strain pulse with a controllable pulse width [108,109,110]. Despite these, the experimental work of strain-mediated 180° magnetization switching without magnetic field assistance in the MTJ architecture is still lacking up to now due to the fabrication difficulties. 

**Figure 11:**
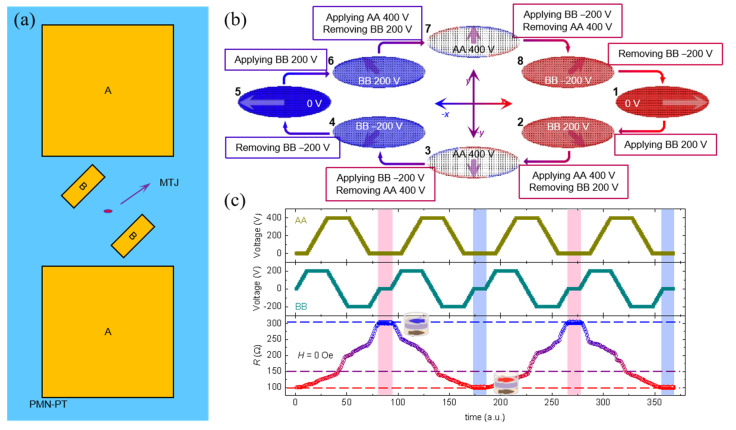
Strain-driven fully switching of TMR by voltage. (**a**) Sketch of the two electrode pairs design in the sample architecture. An MTJ stack was placed at the central area of two electrode pairs. (**b**) Local strain-driven 180° magnetization rotation was achieved via sequential unidirectional 45° rotations by voltage. (**c**) Fully switching process of the MTJ is achieved by exerting successive voltages to the two electrode pairs. Reproduced with permission from reference [113].

It was interesting that Cui et al. [111] reported a scheme by designing a pair of electrodes specially patterned onto a FE substrate to generate localized strain recently. At the central area of the electrode pair, the localized strain is uniform, and the orientation is along the joint line, suggesting the possibility of the strain’s orientation manipulation by specific placing the electrode pair. Furthermore, Biswas et al. [112] patterned two pairs of electrodes, whose joint lines had an angle of 60°, onto the PMN–PT substrate, and the localized strains generated by the two electrodes were noncollinear. After exerting sequential voltages on the two pairs of electrodes, some Co nanomagnets locating at the central area of the electrode pairs rotated 180°, as confirmed by magnetic force microscopy measurements. Chen et al. [113] further developed this scheme in the electric-field control of TMR and replaced the Co nanomagnets with an MTJ stack, as shown in Figure 11a. The joint lines of the two electrode pairs are distanced from the major axis of the elliptical MTJ pillar by 45° and 90°, respectively. The localized strains generated by voltages have two orientations, which is able to rotate the magnetization of the free layer towards the joint line. Then, the alignment of magnetization of the two FM layers can be correspondingly modified due to this rotation. The micromagnetic simulation in Figure 11b clearly reveals that a 180° magnetization rotation can be realized by sequential unidirectional 45° rotations. Consequently, the parallel and antiparallel magnetization configuration could be switched alternatively in the MTJ by this successive voltages to the two electrode pairs, generating a reversible and nonvolatile low/high resistance switching, as shown in Figure 11c. However, the sample architecture becomes more complicated with the introduction of two electrode pairs, which is not conducive to integration and large-capacity memory. Regarding this, this scheme is more superior for applications in lower energy consumption.

## 5. Challenges and Perspectives

The research on ME memory based on strain-mediated FM/FE multiferroic heterostructure is a challenging and significant issue. With the electric-field control of FE domain switching and the in-plane strain in PMN–PT, the magnetic anisotropy of amorphous CFB is tuned correspondingly, generating the modulation of GMR and TMR by electric fields. So far, although the goal of a low-dissipation, nonvolatile, reversible, long-endurance, ME memory operating at room temperature has been realized in the CFB/PMN–PT multiferroic heterostructure, there is still a large number of challenges.

First, the size scalability is still low. A simple design to fully achieve the switching of MTJs between parallel state and antiparallel state, which requires a 180° magnetization rotation of the free layer in MTJ, still remains elusive. Introducing a magnetic interaction to multiferroic heterostructures could be a solution to achieve this goal together with strain coupling, such as exchange interaction [82,83] and dipole interaction [107]. Second, comparing with in-plane MTJs, perpendicular MTJs are more attractive for next generation of high-density memory. Recently, there are large numbers of reports on electric-field control of perpendicular magnetization in FM/FE multiferroic heterostructures with perpendicular magnetic easy axis [114,115,116,117] via strain-mediated ME coupling. Although some of the most spectacular effects have been obtained in the electric-field control of in-plane MTJ, more work is needed to extend these schemes to perpendicular MTJs. Finally, up to now, the MTJs are all deposited on FE PMN–PT substrates. In fact, electric-field control of TMR should be realized in MTJs on the PMN–PT film to achieve integration with current silicon-based electronics. Due to the substrate clamping effect, the piezoelectric property of the FE film is severely reduced, which is not sustainable for strain-mediated ME coupling. Recent emerging freestanding single-crystalline complex-oxide film [34,118,119,120] overcomes the substrate clamping effect and enables modulating magnetism by electric field, which offers a promising way to realize electric-field control of TMR in MTJs on the PMN–PT film.

The error rate is an important feature in the ME memory. The simulation and calculation displayed that the error rate in ME switching was high, but could be reduced via modest theoretical design [121,122,123]. The relevant experimental outcome of error rate is waiting to be verified. In addition, this review constrains the discussion in the static ME coupling. In fact, there is a very new field of time-varying ME coupling, i.e., dynamic ME coupling implemented with surface acoustic waves (SAW) [124], which may further motivate research efforts of moving strain technologies towards memory.

## Data Availability

Data sharing is not applicable to this article.
